# IL-13 induces a bronchial epithelial phenotype that is profibrotic

**DOI:** 10.1186/1465-9921-9-27

**Published:** 2008-03-18

**Authors:** Nikita K Malavia, Justin D Mih, Christopher B Raub, Bao T Dinh, Steven C George

**Affiliations:** 1 Department of Chemical Engineering and Materials Science, University of California – Irvine, Irvine, CA, USA; 2 Department of Biomedical Engineering, University of California – Irvine, Irvine, CA, USA

## Abstract

**Background:**

Inflammatory cytokines (e.g. IL-13) and mechanical perturbations (e.g. scrape injury) to the epithelium release profibrotic factors such as TGF-β_2_, which may, in turn, stimulate subepithelial fibrosis in asthma. We hypothesized that prolonged IL-13 exposure creates a plastic epithelial phenotype that is profibrotic through continuous secretion of soluble mediators at levels that stimulate subepithelial fibrosis.

**Methods:**

Normal human bronchial epithelial cells (NHBE) were treated with IL-13 (0, 0.1, 1, or 10 ng/ml) for 14 days (day 7 to day 21 following seeding) at an air-liquid interface during differentiation, and then withdrawn for 1 or 7 days. Pre-treated and untreated NHBE were co-cultured for 3 days with normal human lung fibroblasts (NHLF) embedded in rat-tail collagen gels during days 22–25 or days 28–31.

**Results:**

IL-13 induced increasing levels of MUC5AC protein, and TGF-β_2_, while decreasing β-Tubulin IV at day 22 and 28 in the NHBE. TGF-β_2_, soluble collagen in the media, salt soluble collagen in the matrix, and second harmonic generation (SHG) signal from fibrillar collagen in the matrix were elevated in the IL-13 pre-treated NHBE co-cultures at day 25, but not at day 31. A TGF-β_2 _neutralizing antibody reversed the increase in collagen content and SHG signal.

**Conclusion:**

Prolonged IL-13 exposure followed by withdrawal creates an epithelial phenotype, which continuously secretes TGF-β_2 _at levels that increase collagen secretion and alters the bulk optical properties of an underlying fibroblast-embedded collagen matrix. Extended withdrawal of IL-13 from the epithelium followed by co-culture does not stimulate fibrosis, indicating plasticity of the cultured airway epithelium and an ability to return to a baseline. Hence, IL-13 may contribute to subepithelial fibrosis in asthma by stimulating biologically significant TGF-β_2 _secretion from the airway epithelium.

## Background

Asthma is a disease characterized by inflammation and chronic repetitive bouts of reversible bronchoconstriction [1]. As the disease progresses there are well-documented structural and phenotypic changes in the airways that have been termed 'airway remodeling'. These structural changes include epithelial damage, goblet cell metaplasia in the airway epithelium, subepithelial fibrosis in the lamina reticularis, smooth muscle cell hyperplasia and hypertrophy, and hyperemia. It is generally thought that these structural changes are the result of inflammation and airway injury and contribute to the chronic progression of the disease. Therapies using corticosteroids and β2 agonists alleviate inflammation and improve pulmonary airflow in mild to moderate asthma; however, their efficacy in reversing structural remodeling in the airways of chronic asthmatics has been limited leading to an impaired quality of life, significant airflow obstruction, bronchial hyperresponsiveness, and decline in lung function [2-10]. The mechanisms underlying these airway structural changes are complex, and only partially understood.

The role of the epithelium in orchestrating subepithelial structural changes in asthma is of keen interest [11-13]. In embryogenesis, the epithelium can dictate mesenchymal differentiation and growth [14]. In asthma, the epithelium is injured in a repetitive fashion, and also exposed to chronic inflammation [15-17]. The result is an altered phenotype which may modulate subepithelial tissue differentiation and growth by influencing the phenotype of numerous neighboring cells including the fibroblast, endothelial cell, and smooth muscle cell through the secretion of various cytokines, chemokines and growth factors [18-20]. Furthermore, most forms of asthma are characterized by abundant Th2 cytokine secretion (e.g IL-4, 5, 9 and, in particular, IL-13), and the over-expression of these cytokines in transgenic mouse models has been shown to reproduce numerous features of asthma including subepithelial fibrosis.

In separate and isolated experiments, it is known that 1) mechanical perturbations and Th2-type cytokine exposure (e.g., IL-13) to the bronchial epithelium can cause the release of profibrotic factors (e.g., TGF-β_2_) [21-29], and induce goblet cell metaplasia [21-23]; and 2) that exogenous TGF-β_2 _stimulates collagen production and secretion from fibroblasts. However, it is not known whether IL-13 can induce phenotypic changes in the airway epithelium which result in TGF-β_2 _secretion at levels that impact collagen secretion and the bulk properties (e.g., optical) of the subepithelial matrix. Furthermore, the ability and time course of the airway epithelium to recover from a repeated inflammatory insult and return to a baseline phenotype has not been described. Finally, over expression of IL-13 has also been implicated in the development of other fibrotic diseases including idiopathic pulmonary fibrosis (IPF) [24]. An impaired signaling between the epithelium and stroma has been suggested, however these mechanisms are only partially understood [25,26].

We hypothesized that phenotypic changes induced by IL-13 create an epithelium that is profibrotic; that is, an IL-13-treated epithelium could secrete soluble mediators in a continuous fashion to induce changes in a subepithelial fibroblast-embedded matrix consistent with fibrosis, but in the absence of the Th2 cytokine. Using a co-culture model of fully mucociliary-differentiated normal human bronchial epithelial cells and normal human lung fibroblasts embedded in a collagen gel [27-29], we found that prolonged (14 days) exposure to IL-13 during the differentiation phase induced an increase in MUC5AC expression which persisted for up to seven days following withdrawal of IL-13. Furthermore, this altered epithelial phenotype stimulated soluble collagen release in the media, increased deposition of salt soluble collagen in the matrix, and enhanced the second harmonic generation (SHG) signal from fibrillar collagen in the subepithelial matrix. The enhanced collagen content and changes in the optical properties are due, in part, to the continuous secretion of epithelial-derived TGF-β_2_. Furthermore, the return to a baseline phenotype of the airway epithelium was observed following withdrawal of IL-13 for a period of ten days, demonstrating plasticity of the airway epithelial phenotype.

## Methods

### Materials

Recombinant human IL-13 (Cat # 213-IL), TGF-β_2 _(Cat # 302-B2), and polyclonal goat TGF-β_2 _neutralizing antibody (Cat # AB-112-NA) were purchased from R&D Systems (Minneapolis, MN). Human active and total TGF-β_2 _was measured in the media using ELISA per manufacturers instructions (Cat # DB250, R&D Systems). Sircol™ soluble collagen assay was obtained from Accurate Chemical (Westbury, N.Y., USA) and performed following the manufacturers instructions. Purified goat IgG and all other chemicals were purchased from Sigma (St. Louis, MO).

### Cell culture

Cryopreserved passage 1 normal human bronchial epithelial (NHBE) cells from three different donors (donor 1: 4F1624, donor 2: 4F1430, donor 3: 5F1387) were obtained from Lonza (formerly Cambrex, Walkersville, MD). Cells were thawed and passaged twice in T-75 cm^2 ^flasks (Corning, Fisher) in a 37°C, 5% CO_2_/95% air incubator in bronchial epithelial growth medium (BEGM) supplemented with growth factors supplied in the SingleQuot^® ^kit (Lonza). Cells were trypsinized and seeded (day 0) at passage 3 onto uncoated Costar Transwells^® ^inserts with 0.4 μm pore size (Corning, Fisher) at a density of 1.5 × 10^5 ^cells/cm^2 ^in media comprised of 50% BEBM and 50% DMEM-F12 low glucose (Invitrogen). This media was then supplemented with the growth factors provided in the SingleQuot^® ^kits and retinoic acid at 50 nM as previously described [27], and will be referred to as "50:50 media".

Once the cells were confluent (approximately seven days after seeding), they were switched to an air-liquid interface for 2 weeks (days 7–21) to achieve mucociliary differentiation. In some cases, a varying concentration of IL-13 (0.1, 1 or 10 ng/ml) was added to the basal medium from day 7 to day 21(14 day treatment). In vivo IL-13 concentrations are not known; thus, we selected concentrations based on previous reports that induced mucus cell metaplasia in the bronchial epithelium [21,22,30-33].

Primary normal human lung fibroblasts (NHLF, Lonza) were used between passage 3 and 7. Cells were routinely cultured as a monolayer in T-75 cm^2 ^flasks (Corning, Fisher) in fibroblast growth media (FGM-2) supplemented with growth factors supplied in the corresponding SingleQuot^® ^kit (Lonza). The NHLF were seeded in 1.7 mg/ml rat-tail tendon collagen 1 (Collaborative, Bedford, MA), 5× DMEM (Gibco, Invitrogen) and 10× reconstitution buffer (25 mM NaHCO_3_, 20 mM HEPES, and 5 mM NaOH). 800 μl aliquots of the mixture containing 25,000 cells per ml were pipetted into 12 well plates, and some into glass bottom plates specially designed for Confocal microscopy (Mattek Corp.). The collagen mixture was then non-covalently crosslinked in 5% CO_2 _for 1 h at 37°C, after which 1 ml of 50:50 media was added to each well.

**Figure 1:**
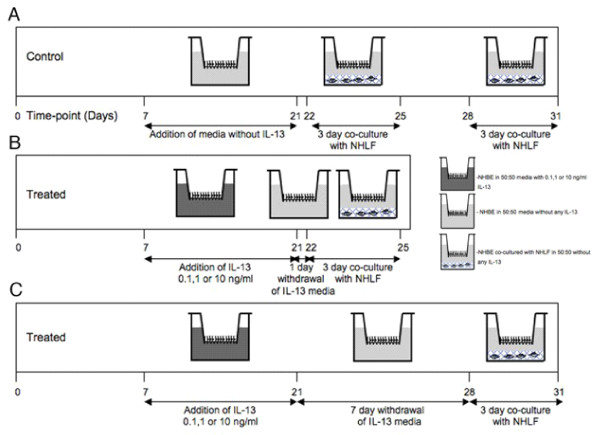
**Protocol for IL-13 treatment and withdrawal.** NHBE cells are seeded on Transwells^® ^as described in the *Materials and Methods*, treatment with IL-13 followed by its withdrawal is carried out as shown. (A) For the control case, the NHBE are cultured in 50:50 epithelial media without any IL-13 all throughout and co-cultured with NHLF embedded in rat tail collagen gel from days 22 to day 25 and day 28 to day 31. (B) NHBE are treated for 14 days from day 7 to day 21 with varying IL-13 concentrations (0.1, 1, 10 ng/ml), then the IL-13 media is withdrawn and replaced with 50:50 epithelial media for 1 day from day 21 to day 22. On day 22 the NHBE are co-cultured with NHLF embedded in a rat-tail collagen gel for a period of 3 days till day 25. (C) The NHBE are treated for 14 days from day 7 to day 21 with varying IL-13 concentrations (0.1, 1, 10 ng/ml), then the IL-13 media is withdrawn and replaced with 50:50 media for a period of 7 days from day 21 to day 28. On day 28 the NHBE are co-cultured with NHLF embedded in a rat-tail collagen gel for 3 days till day 31.

Fig. 1 describes the different experimental conditions schematically. After the 14 days treatment with varying IL-13 concentrations, in one group (Fig. 1B), IL-13 media is withdrawn (removed) at day 21. At day 22, some of these Transwells^® ^were moved to new 12 well plates and co-cultured with NHLF embedded in rat tail collagen gels for 3 days (days 22–25) in 50:50 media without IL-13, with/without TGF-β_2 _neutralizing antibody or goat IgG control. In a second group (Fig. 1C), the IL-13 media was withdrawn at day 21 and cultured in 50:50 media without IL-13 until day 28, and then co-cultured with NHLF embedded rat tail collagen gels for 3 days (days 28–31), with/without TGF-β_2 _neutralizing antibody or goat IgG control. A third group served as the control (Fig. 1A), where the co-cultures were performed at the same time points, but the cells were never exposed to IL-13. A final condition consisted of NHLF-embedded collagen gels alone (not co-cultured with NHBE) with 1 ml of 50:50 media on top, and is referred to as "NHLF only". Media was collected from all conditions and stored at -80°C for active and total TGF-β_2 _analysis by ELISA. A critical feature of the study design is treatment of the NHBE with IL-13 for 14 days followed by withdrawal and subsequent culture in the absence of IL-13 all throughout the withdrawal and 'co-culture with NHLF' periods.

### Immunofluorescence microscopy

At day 22 (i.e. after 14 days IL-13 treatment and 1 day withdrawal of IL-13 containing media) and day 28 (i.e. 14 day treatment and 7 day withdrawal of IL-13 media), the NHBE were fixed using 4% formaldehyde (Sigma) in PBS at 4°C for 20 minutes. Non-specific binding was blocked by addition of Abdil (2% BSA in TBS-0.1% Triton-X) for 1 hr at 4°C. Samples were incubated in mouse monoclonal anti-MUC5AC (Clone 45M1, Neomarkers, Fremont, CA, diluted 1:500 in Abdil) or anti-β-Tubulin IV (Sigma Aldrich, St. Louis, MO, diluted 1:1000 in Abdil) overnight followed by wash and incubation with Alexa Fluor 488 anti-mouse secondary antibody (Molecular probes, Eugene, OR) at 1:500 in Abdil for 2 hr at 4°C. Cell nuclei were stained with 4', 6-diamidino-2-phenylindole dihydrocholride hydrate (1 μg/ml, DAPI, Sigma) in PBS for 5 minutes. After staining, the Transwell^® ^membrane was removed by a scalpel, placed on a microscope slide with a drop of Vectashield, and visualized using a Nikon Eclipse E800 epifluorescence microscope.

### SDS-PAGE and western blot

At day 22, 28, 25 and 31 some of the NHBE monolayers alone or from co-cultures were lysed using RIPA buffer on ice (50 mM Tris, pH 8.0, 150 mM NaCl, 1% Nonidet P-40, 0.1% SDS, 0.5% sodium deoxycholate, and 0.1 mM sodium orthovanadate) supplemented with protease inhibitor cocktail (Sigma, P8340) by repetitive scraping. Protein concentrations were determined using BCA protein assay (Pierce Biotechnology) following the manufacturers directions. Laemmli buffer was added to 40 μg equal protein in gel running buffer and then boiled for 5 minutes. Samples were subjected to SDS-PAGE and transferred onto nitrocellulose (0.1 A, overnight). Western blotting was performed using appropriate primary (monoclonal mouse anti-MUC5AC or anti-β-Tubulin-IV were diluted in 5% milk in TBS-0.1% Tween at 1:500 and 1:1000 respectively) and horseradish peroxidase (HRP) conjugated secondary antibodies (1:10,000, Santa Cruz biotechnology), and visualized using an enhanced chemiluminescence system (Amersham Biosciences and Biorad Imaging system). The blots were also probed with mouse monoclonal anti-β-actin (Abcam) as a loading control.

### Sircol™ soluble collagen assay

At day 25 and day 31, collagen gels from the co-culture and NHLF only conditions were collected in different 15 ml centrifuge tubes (6 gels per condition, per time point from two donors), weighed and stored at -80°C. At the start of the experiment for extracting salt soluble collagen, 0.05 M Tris buffer (pH 7.5) containing 1 M NaCl with 1:100 protease inhibitor cocktail (P8340, Sigma) was added to the gels. A ratio of 5 volumes of solvent to wet tissue weight was used. The sample was then stirred overnight at 4°C. The next day they were centrifuged at 1,000 g for 5 minutes to obtain a colorless supernatant. Sircol™ soluble collagen assay was then performed following the manufacturers directions. In brief, 1 ml of Sircol dye was added to 200 μl of supernatant extracted from collagen gels, as described above, or 200 μl of cell culture media. The tubes were mixed on a shaker for 30 minutes and then centrifuged at 10,000 g for 10 minutes to obtain a well-compacted pellet composed of precipitated collagen-dye complex, followed by inverting and draining the tubes on tissue paper to remove unbound dye. 1 ml of alkali reagent was added to the pellet in each tube, and the tubes were thoroughly vortexed to dissolve the bound dye pellet. 200 μl of this mixture for the sample (either extracted from collagen gels or cell culture media), a corresponding blank and standard (conc. 1 mg/ml used to generate a standard curve, supplied in the Sircol assay kit) was then analyzed in triplicate using a 96-well plate in a spectrophotometer at 540 nM in a Benchmark Microplate Reader (Biorad). All values are expressed as a percentage of NHLF embedded in rat-tail collagen gels levels ("NHLF only").

### Multiphoton microscopy

Some co-cultures were developed in 12 well glass bottom plates (Mattek Corp.) for imaging the NHLF-embedded collagen gels at day 25 and day 31. The network of collagen fibers was studied in the extracellular matrix of the co-culture model using multiphoton microscopy (MPM) as previously described [34,35]. Briefly, a Zeiss LSM 510 Meta multiphoton microscope (Zeiss, Jena, Germany) was used. A Mai Tai laser was tuned to 780 nm and focused on the sample with an EC Plan-Neofluar 40×/1.3 NA oil DIC objective (Zeiss). Power before the objective was 250 mW. Resolution was ~0.4μm in x-, y- and 1 μm in z- image planes, with an area of 0.019 μm^2 ^per pixel. Each image was 512 × 512 pixels. Pixel intensity histograms showed minimal pixel saturation. The meta channel was used to collect emitted light in an epiconfiguration at wavelength 390 nm using a narrow bandpass filter (380–400 nm), which exclusively represented the second harmonic generation (SHG) signal from fibrillar collagen [36,37]. Image stacks were collected at 10-micron intervals between 20–120 microns from the coverslip. Three image stacks were collected per gel at random locations with each stack at least 1 mm apart, and for three gels per condition and per timepoint. The LSM 510 software (Zeiss) was used to quantify images using the average segmented pixel intensity.

### Statistics

Experiments were performed using three NHBE donors, repeated twice, with 3–6 wells/gels per condition per time point. Data are reported as mean ± SD and InStat 2.01 for Macintosh software package was used for all analysis. Data were analyzed using one-way analysis of variance (ANOVA) with Student Newman Keuls post-test analysis for multiple comparisons. Data were considered significant at p < 0.05.

## Results

### IL-13 treatment and plasticity of the NHBE

**Figure 2:**
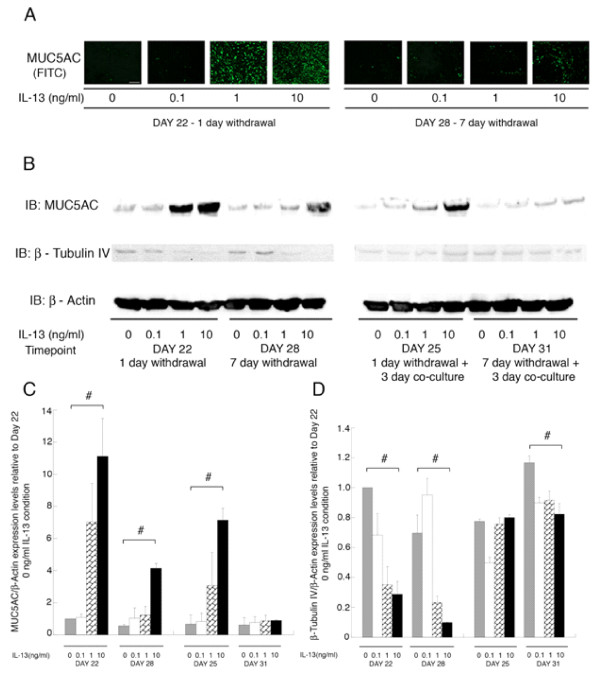
**IF staining and Western blot for MUC5AC/β-Tubulin IV in the NHBE.** IL-13 mediated a concentration dependent increase in MUC5AC protein levels in the NHBE as seen by (A) Immunofluorescence, where at day 22 and day 28, 1 and 7 days after withdrawal of 14 day treatment with IL-13 (1,10 ng/ml for day 22 and 10 ng/ml for day 28) the staining for MUC5AC is higher compared to the untreated NHBE (0 ng/ml IL-13) (n = 3 donors of NHBE; grown in duplicate; with 3–6 wells per condition; scale bar = 20 μm). DAPI staining of the nuclei showed similar number of cells in all conditions (data not shown). (B) Levels of MUC5AC protein show a dose dependent increase via western blot at day 22 and day 28. Also during co-culture with the NHLF the dose dependent increase of MUC5AC is maintained at day 25 and not at day 31. Levels of β-Tubulin IV protein in the NHBE shown an inverse dependence on IL-13 concentration at days 22 and day 28 with levels remaining constant at day 25 and day 31 of co-culture with NHLF. Images are representative from 3 NHBE donors. (C, D) Quantification of MUC5AC/β-Actin and β-Tubulin IV/β-Actin levels relative to IL-13 concentration of 0 ng/ml at day 22 condition, show a dose dependent increase with IL-13 concentration at day 22,28 and 25 for MUC5AC and dose dependent decrease at day 22, 28 and 31 for β-Tubulin IV (Statistical difference between conditions by ANOVA # p < 0.01).

At day 22, after 14 days treatment with 1 or 10 ng/ml of IL-13 followed by 1-day withdrawal of IL-13, the NHBE demonstrate a increase in MUC5AC protein as detected by immunofluorescence (Fig. 2A). At day 28, when the IL-13 treatment has been withdrawn for 7 days, the treated NHBE (10 ng/ml) still demonstrate elevated levels of MUC5AC over the untreated control (i.e. NHBE cultured in media without any IL-13) condition (Fig. 2A). Chronic treatment with the lowest concentration (0.1 ng/ml) of IL-13 did not increase MUC5AC staining over control (0 ng/ml IL-13) either at day 22 or day 28 (Fig. 2A).

Immunoblotting for MUC5AC protein mirrored the trends of immunofluorescence at days 22 and day 28. At day 25 (1 day withdrawal of IL-13 treatment followed by 3 day co-culture with NHLF), MUC5AC protein by immunoblot increased in a dose-response fashion with IL-13 treatment concentration (Fig. 2B, C). In contrast, by day 31 (7 day withdrawal of IL-13 treatment followed by 3 day co-culture with NHLF) the MUC5AC protein levels in NHBE for all treatment concentrations were no different than control levels.

The trends in the protein level of β-Tubulin-IV are the opposite of MUC5AC (Fig. 2B, D). As IL-13 exposure concentration increases, the amount of β-Tubulin-IV decreases. This effect of IL-13 is observed both 1-day (day 22) and 7-days (day 28) following withdrawal of the IL-13, but is not observed in the conditions following 3-days of co-culture with the NHLF (days 25 and 31). Images are from one representative donor.

### IL-13 stimulates TGF-β_2 _release from NHBE

**Figure 3:**
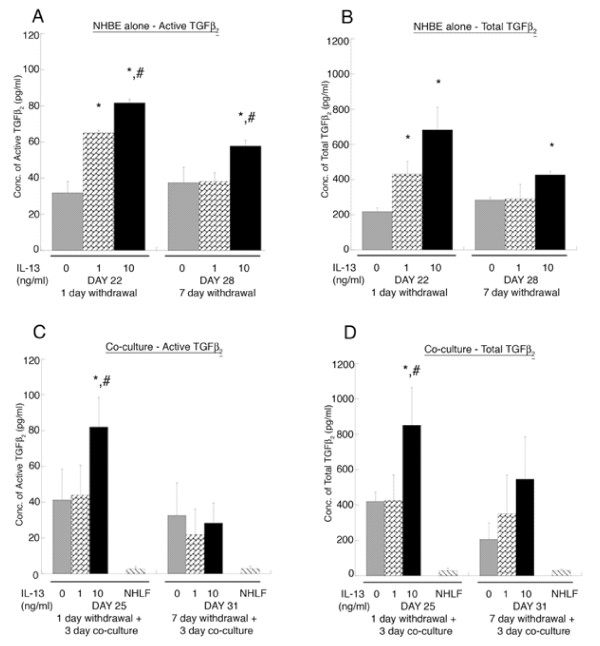
**ELISA for active and total TGF-β_2 _in the media (A, B).** At day 22, the concentration of active and total TGF-β_2 _in the media of IL-13 pre-treated NHBE at 1 and 10 ng/ml is significantly higher as compared to untreated NHBE (0 ng/ml of IL-13) media; * p < 0.01. At day 22 and day 28, the concentration of active TGF-β_2 _in the IL-13 pre-treated NHBE at 10 ng/ml is elevated compared to pre-treated NHBE at 1 ng/ml; # p < 0.01. At day 28 active and total TGF-β_2 _in IL-13 pre-treated NHBE at 10 ng/ml is increased compared to untreated NHBE; * p < 0.01. (C, D) At day 25, the NHBE pre-treated with IL-13 at 10 ng/ml, has higher levels of active and total TGF-β_2 _in the media as compared to untreated and pre-treated NHBE at 1 ng/ml co-cultured with NHLF (*, # p < 0.01 compared to 0 and 1 ng/ml IL-13 pre-treated NHBE co-cultured with NHLF, respectively). At day 31, there is no significant difference in the levels of active and total TGF-β_2 _between treatment conditions. NHLF represents levels of active and total TGF-β_2 _in media of fibroblasts in a collagen gel without NHBE co-culture. All experiments were performed using 3 donors, grown in duplicate, with 3–6 wells per condition.

Fig. 3A, B demonstrates that the IL-13 treatment induces active and total TGF-β_2 _release from the airway epithelium in the media (as measured by ELISA) over baseline levels secreted from the untreated NHBE and the 10 ng/ml pre-treated NHBE levels remain elevated at day 28 (7 days post withdrawal of IL-13 treatment). Fig. 3C–D shows that active and total TGF-β_2 _release remains significantly (p < 0.01) elevated at day 25, following 14 days IL-13 treatment at 10 ng/ml, 1 day withdrawal and co-culture with NHLF, although at day 31 following 7 day withdrawal after treatment, the levels of TGF-β_2 _are no different from control (i.e. untreated NHBE co-cultured with NHLF). NHLF embedded in a rat tail collagen gel, "NHLF only" (Fig. 3C–D) secrete negligible levels of active and total TGF-β_2_. Chronic treatment with the lowest concentration (0.1 ng/ml) of IL-13 did not increase levels of active and total TGF-β_2 _over control at any of the time points and thus was not used in the following experiments.

### IL-13 treated epithelium secretes biologically relevant TGF-β_2 _that stimulates collagen secretion

**Figure 4:**
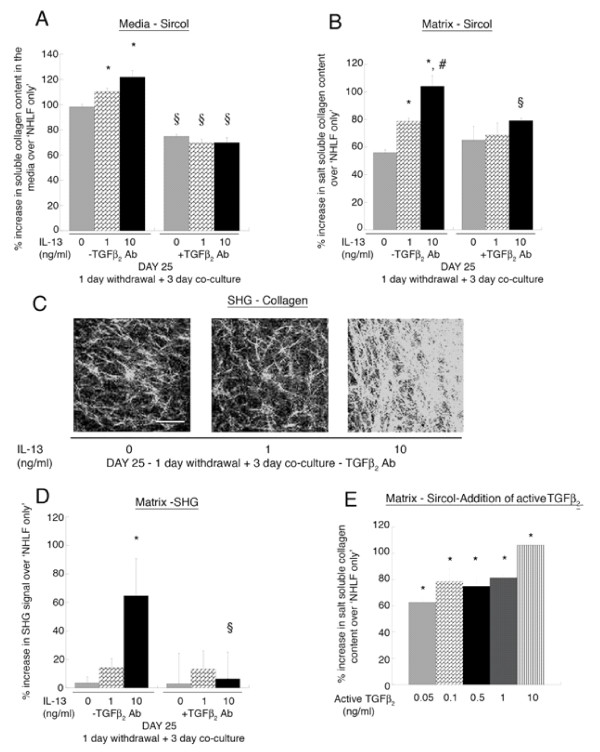
**Quantification of soluble collagen content in the media and matrix.** (A) Sircol soluble collagen assay was performed as described in the *Materials and Methods*, which quantifies the amount of soluble collagen in the cell culture supernatant and newly synthesized salt soluble collagen in the matrix. The amount of soluble collagen secreted in the media at day 25 in the IL-13 pre-treated NHBE at 1 and 10 ng/ml co-cultured with NHLF is augmented as compared to the untreated NHBE co-culture; * p < 0.01 and addition of TGFβ_2 _neutralizing antibody (10 μg/ml) abolishes this increase (^§ ^p < 0.01 compared to respective condition without TGFβ_2 _neutralizing antibody). (B) At day 25 there is an increase in newly synthesized salt soluble collagen content in the matrix in the IL-13 pre-treated NHBE at 1 and 10 ng/ml followed by co-culture with NHLF as compared to the untreated NHBE co-culture; * p < 0.01 and the IL-13 pre-treated NHBE at 10 ng/ml co-culture collagen levels are elevated as compared to the IL-13 pretreated NHBE at 1 ng/ml co-culture; # p < 0.01. Also, addition of the TGFβ_2 _neutralizing antibody abolishes this increase (^§ ^p < 0.01 compared to respective condition without TGFβ_2 _antibody). The media and matrix collagen levels are normalized to respective levels obtained from NHLF embedded in collagen gels ("NHLF only"). (C, D) Representative Second harmonic generated (SHG) images (scale bar = 50 μm) of collagen fibrils at day 25 are shown along with the quantification of signal intensities. The SHG signals from the collagen secreted by NHLF embedded in rat tail collagen gels which were co-cultured with the IL-13 pre-treated NHBE at 10 ng/ml are elevated compared to the untreated NHBE co-culture; * p < 0.01 and this increase is inhibited on incubation with TGFβ_2 _neutralizing antibody in the 3 day co-culture period (^§ ^p < 0.01 compared to respective condition without TGFβ_2 _antibody). Addition of goat IgG did not alter the increased levels of collagen in the matrix and media in the pre-treated NHBE-NHLF co-culture. (E) Exogenous active TGF-β_2 _at 0.05, 0.1, 0.5, 1 and 10 ng/ml is added in 50:50 epithelial media to NHLF embedded in collagen gels for a period of 3 days. There is a significant increase in the newly synthesized salt soluble collagen content in the matrix with addition of increasing concentration of active TGF-β_2 _(* p < 0.01 compared to only NHLF condition). All values are normalized to those obtained from "NHLF only" condition. All experiments were performed using 3 donors, grown in duplicate, with 3–6 wells for each condition.

To investigate further the physiological relevance of epithelial-derived TGF-β_2 _on collagen secretion from the NHLF and its ability to modulate the optical properties of the matrix, we co-cultured pre-treated and untreated NHBE with NHLF embedded in collagen gels. At day 25 the NHLF embedded collagen gels demonstrate significantly (p < 0.01) elevated levels of soluble collagen secreted in the media (Fig. 4A) and matrix (Fig. 4B), in the co-culture of NHBE pre- treated with 1 or 10 ng/ml IL-13 as compared to control (i.e. untreated NHBE co-cultured with NHLF). This increase is abolished on incubation with TGF-β_2 _neutralizing antibody (10 μg/ml) in the 3-day co-culture period (levels were unaffected upon incubation with purified goat IgG control). Furthermore the SHG signal (Fig. 4C–D), which is an index of collagen fibril organization and density from the matrix (20 μm from the surface), is augmented from the 10 ng/ml IL-13 pre-treated NHBE-NHLF co-culture. This increase is also inhibited upon incubation with TGF-β_2 _neutralizing antibody (10 μg/ml). At day 31 there is no significant difference in the levels of collagen secreted or SHG signal from the IL-13 treated and untreated NHBE co-cultures (data not shown).

Exogenous active TGF-β_2 _at 0.05, 0.1, 0.5, 1, and 10 ng/ml (Fig. 4E) was added in epithelial media to NHLF embedded in collagen gels for a period of 3 days and collagen secretion assayed. The amount of collagen secreted in the matrix as compared to "NHLF only" was increased, in a dose-dependent manner. All values are expressed as a percentage of NHLF embedded in rat-tail collagen gels levels ("NHLF only").

## Discussion

Asthma affects 8%–10% of the population, and is characterized by chronic airway inflammation, repetitive bronchoconstriction, and marked structural changes in the airway wall including goblet cell metaplasia in the airway epithelium, and collagen deposition in the lamina reticularis (sub-epithelial fibrosis) [9,38,39]. Furthermore, other fibrotic diseases in the lungs (e.g., IPF) share similar characteristics [24-26,40]. Mechanisms linking these features of the disease are only partially understood. Our study demonstrates that a prominent TH2-type inflammatory mediator, IL-13, can alter the differentiated phenotypic features of the epithelium. Furthermore, the altered epithelium alone (i.e., in the absence of IL-13) secretes biologically significant TGF-β_2 _levels, which stimulates features of fibrosis (e.g., collagen secretion) in the subepithelial matrix and alters the bulk optical properties of the matrix. In addition, following extended withdrawal of IL-13, the epithelium is capable of reverting back to its baseline phenotype.

The inflammatory process in asthma has a prominent allergic component which involves Th2-type cytokines including interleukin(IL)-4, -5 and -13 [1]. Both in vivo and in vitro model systems have been employed to determine the role of IL-13 in modulating features of the disease [41,42]. In vivo, transgenic mice which selectively over express IL-13 or mice which do not express IL-13 have been used to demonstrate critical roles of IL-13 in airway hyperresponsivness, fibrosis, and mucus cell metaplasia [43,44]. However, the source of IL-13 leading to these findings cannot be isolated as multiple cells types are capable of producing IL-13. IL-13 mediated changes to the bronchial epithelium could be paracrine (Th2 lymphocytes as the source) or autocrine (epithelium as the source) [45].

In vitro, treatment of the airway epithelium with IL-13 during the differentiation phase has been shown to stimulate mucus production [22,23,30] and acute (48 hours) treatment of the epithelium with IL-13 can stimulate the release TGF-β_2 _[32,33]. Both of these observations are consistent with our results. Nonetheless, the biological consequence(s) of these IL-13-induced changes to the epithelium have not been described.

The current study demonstrates that chronic treatment of the epithelium with IL-13 during the differentiation phase results in enhanced MUC5AC expression (Fig. 2A–C), reduced β-Tubulin-IV expression (Fig. 2B, D) and elevated TGF-β_2 _secretion (Fig. 3A–D). These changes are observed for up to seven days following withdrawal of the IL-13. When the IL-13 pre-treated epithelium was co-cultured with a fibroblast-embedded collagen gel in the absence of IL-13 at day 25, the IL-13 concentration dependent increase in MUC5AC was maintained but not at day 31. However, the down regulation of β-Tubulin-IV expression with increasing IL-13 concentration was suppressed during the co-culture with fibroblasts at day 25, and this trend was also observed at day 31. While we did not pursue the mechanism of this observation at this time, it seems clear that the fibroblasts influence the epithelium through as yet unidentified mediators. This observation lends support to co-culture models as they provide unique insight into epithelial and mesenchyme communication.

Although there is some donor to donor variability, the trends for all these proteins remain the same [46]. In addition, IL-13 can stimulate cell proliferation [31], which may account for increased levels of TGF-β_2 _and MUC5AC, we did not observe a significant increase in cell number on staining nuclei (data not shown). These phenotypic changes are consistent with the loss of ciliated epithelial cells (reduced β-Tubulin-IV), and goblet cell metaplasia (enhanced expression of MUC5AC).

At day 25, when the IL-13 treated epithelium was co-cultured with a fibroblast-embedded collagen gel in the absence of IL-13, the fibroblasts increased both the secretion of soluble collagen in the media and matrix (Fig. 4A–B), and the second harmonic generated signal in the extracellular matrix (Fig. 4C–D). Upon incubation with a TGF-β_2 _neutralizing antibody this increase is abolished suggesting that biologically relevant levels of TGF-β_2 _levels are continuously secreted by the epithelium. Furthermore, at day 31 when the levels of TGF-β_2 _are the same in the treated and untreated NHBE co-cultures, there is no increase in collagen content in the media or matrix condition nor were there any differences in the SHG signals.

Thickening of the reticular layer in asthma has been termed subepithelial fibrosis, and is due to deposition of fibrillar collagens (types I, III, and V), tenascin C, and fibronectin [9]. The Sircol collagen assay is a quantitative dye-binding method that can measure collagens from type I-V in a soluble form. Only newly secreted collagen into the matrix is soluble. Over time, collagen becomes insoluble due to intermolecular crosslinking. Our results demonstrate at day 25 that soluble collagen levels are elevated when the fibroblast-embedded collagen gel is co-cultured with the airway epithelium compared to levels without co-culture, and an additional increase when the airway epithelium has been differentiated in the presence of IL-13. This observation is consistent with enhanced collagen secretion by the fibroblasts due to soluble mediators produced by the airway epithelium [28,29,47,48]. It has been shown that IL-13 can induce the secretion of matrix metalloproteases that could potentially degrade the rat-tail collagen gels. We tested this possibility by taking the media at day 25 and day 31 from the varying concentration IL-13 pretreated NHBE-NHLF co-culture conditions and exposing it to acellular collagen gels for 3 days. We did not observe a significant change in the levels of collagen in the media before and after exposure to the gels (data not shown), suggesting that, although IL-13 may induce secretion of matrix metalloproteases, the type or magnitude did not impact or degrade the rat tail collagen in our system.

In addition to the Sircol soluble collagen assay, we quantified structural changes in the matrix using multiphoton microscopy and second harmonic generation (SHG). SHG in the extracellular matrix is specific to fibrillar collagen, and is generated by non-linear interactions of the near-infrared light with the non-centrosymmetric features of collagen. SHG is largely forward propagated from collagen fibers at exactly 1/2 the wavelength of the excitation wavelength [35,49]. In our experiment, we utilized an excitation wavelength of 780 nm, and detected the subsequently backward scattered SHG signal (390 nm) from collagen using a narrow bandpass filter (380–400 nm) in an epiconfiguration. The intensity of the SHG signal is a positive function of the concentration of collagen, but can also increase when the organization of collagen at secondary and tertiary levels increases [37]. Thus, at day 25 our observation of an enhanced SHG signal in the extracellular matrix following co-culture of the airway epithelium with the fibroblast-embedded collagen gel is consistent with either an increased amount of collagen, and/or an increased organization of the collagen.

We hypothesized that the increase in collagen secretion and the enhanced SHG signal from the matrix was due, in part, to epithelial-derived TGF-β_2_. TGF-β has three isoforms (TGF-β_1_, TGF-β_2_, TGF-β_3_) in mammalian systems [50], and are pleiotropic mediators of cell growth and differentiation [51]. All three isoforms are present in the lungs, can be produced by epithelial cells, and have been shown to contribute to fibrosis. For example, our group has recently demonstrated that scrape-injured airway epithelial cells release active TGF-β_2 _at concentrations similar to the current study (50–100 pg/ml), and that the epithelial-derived TGF-β_2 _enhances the SHG signal from an underlying fibroblast-embedded collagen gel [28]. Moreover, relatively small deviations (i.e., 30–50 pg/ml) above or below the basal production of TGF-β_2_, that are similar in magnitude observed in our study, result in altered SHG from the matrix suggesting that tight regulation of TGF-β_2 _is required for normal matrix homeostasis. Similarly, it has been shown that compression of airway epithelial cells stimulates collagen secretion from fibroblasts in co-culture [47], as well as TGF-β_2 _release [29]. The role of biologically relevant epithelial-derived TGF-β_2 _in subepithelial fibrosis is also strongly supported by our observation that a neutralizing antibody negates the observed increases in collagen secretion and SHG, and that addition of exogenous active TGF-β_2 _(Fig. 4E) at concentrations observed in the media reproduces the increase in collagen secretion.

TGF-β_1 _was measured in the media in our co-culture model (60–70 pg/ml, data not shown), but the concentration was not impacted by IL-13 treatment or co-culture. Transgenic mice bred to over express IL-13 demonstrate tissue fibrosis and stimulate TGF-β_1 _production. Although the major source of TGF-β_1 _in the mouse model are macrophages, alveolar epithelial cells, and eosinophils, we cannot rule out the role of TGF-β_1 _in subepithelial fibrosis [52,53].

Finally, the normal human bronchial epithelial cells are commercially (Lonza) purchased primary cells from normal human healthy and non-smoking individuals who are tested and found non-reactive by an FDA approved method for the presence of HIV-1 and other viruses. The asthmatic airway epithelium in vivo displays signs of structural damage, it is more susceptible to oxidant-induced apoptosis and has marked mucus metaplasia. It is widely accepted that the epithelium in asthmatics is biochemically abnormal due to its ability to release greater amounts of pro-inflammatory cytokines and express elevated levels of transcription factors both in vivo and following in vitro culture [12,54,55]. Thus, the asthmatic airway epithelium may respond differently to IL-13 than the normal airway epithelium; nonetheless, the current study forms the basis for observing similar endpoints in future studies using asthmatic epithelial cells.

## Conclusion

IL-13 enhances MUC5AC expression and TGF-β_2 _secretion, and decreases β-Tubulin-IV expression in the airway epithelium when present during the 14-day differentiation phase at an air-liquid interface. The altered phenotypic features of the airway epithelium in vitro are consistent with those observed in asthma. Co-culturing this altered epithelial phenotype with a lung fibroblast-embedded collagen gel in the absence of IL-13 results in enhanced collagen secretion and second harmonic generation signal from the extracellular matrix, both of which are dependent on biologically significant levels of epithelial-derived TGF-β_2_. However, upon withdrawal for a period of ten days, the levels of MUC5AC, β-Tubulin-IV and TGF-β_2 _secretion are similar in the treated and untreated case indicating plasticity of the cultured airway epithelium, and its ability to return to a baseline phenotype. We conclude that IL-13 may contribute to subepithelial fibrosis in asthma by stimulating the continuous release TGF-β_2 _from the airway epithelium.

## Competing interests

The author(s) declare that they have no competing interests.

## Authors' contributions

NKM designed, planned, and performed all of the experiments, and wrote the manuscript; JDM performed some initial studies regarding NHBE culture and IL-13 exposure; CBR and BTD assisted with the design and interpretation of multiphoton microscopy and SHG imaging; and SCG provided overall guidance for the study, assisted in the experimental design, analysis and interpretation of the data, and writing of the manuscript. All authors have read and approved the final manuscript.
